# Levels of depression, anxiety, and psychological distress among Ugandan adults during the first wave of the COVID-19 pandemic: cross-sectional evidence from a mobile phone-based population survey

**DOI:** 10.1017/gmh.2022.28

**Published:** 2022-06-30

**Authors:** Emma Clarke-Deelder, Slawa Rokicki, Mark E. McGovern, Catherine Birabwa, Jessica L. Cohen, Peter Waiswa, Catherine Abbo

**Affiliations:** 1 University of Basel, Basel, Switzerland; 2 Department of Epidemiology & Public Health, Swiss Tropical & Public Health Institute, Allschwil, Switzerland; 3 Department of Global Health & Population, Harvard T. H. Chan School of Public Health, Boston, Massachusetts, USA; 4 Department of Health Behavior, Society, and Policy, Rutgers School of Public Health, Piscataway, New Jersey, USA; 5 Geary Institute for Public Policy, University College Dublin, Dublin, Ireland; 6 Department of Health Policy, Planning and Management, Makerere University School of Public Health, Kampala, Uganda; 7 Global Public Health, Karolinska Institute, Stockholm, Sweden; 8 Department of Psychiatry, Makerere University College of Health Sciences, Kampala, Uganda

**Keywords:** Anxiety, COVID-19, depression, global health, low and middle-income countries, population mental health, psychological distress

## Abstract

**Objectives:**

Policy measures to slow the spread of coronavirus disease 2019 (COVID-19), such as curfews and business closures, may have negative effects on mental health. Populations in low- and middle-income countries (LMICs) may be particularly affected due to high rates of poverty and less comprehensive welfare systems, but the evidence is scarce. We evaluated predictors of depression, anxiety, and psychological distress in Uganda, which implemented one of the world's most stringent lockdowns.

**Methods:**

We conducted a mobile phone-based cross-sectional survey from December 2020 through April 2021 among individuals aged 18 years or over in Uganda. We measured depression, anxiety, and psychological distress using the Patient Health Questionnaire (PHQ)-2, the Generalized Anxiety Disorder (GAD)-2, and the PHQ-4. We applied linear regression to assess associations between experiences of COVID-19 (including fear of infection, social isolation, income loss, difficulty accessing medical care, school closings, and interactions with police) and PHQ-4 score, adjusted for sociodemographic characteristics.

**Results:**

29.2% of 4066 total participants reported scores indicating moderate psychological distress, and 12.1% reported scores indicating severe distress. Distress was most common among individuals who were female, had lower levels of education, and lived in households with children. Related to COVID-19, PHQ-4 score was significantly associated with difficulty accessing medical care, worries about COVID-19, worries about interactions with police over lockdown measures, and days spent at home.

**Conclusions:**

There is an urgent need to address the significant burden of psychological distress associated with COVID-19 and policy responses in LMICs. Pandemic mitigation strategies must consider mental health consequences.

## Introduction

The coronavirus disease 2019 (COVID-19) pandemic is a global public health crisis of many dimensions. In addition to the enormous burden of morbidity and mortality, policy responses to the pandemic, including lockdowns, curfews, and school and business closures, have resulted in enforced isolation, unemployment and financial strain, and disruptions in daily life and social support (Campion *et al*., 2020; Kola *et al*., 2021). There is mounting evidence that the pandemic has significantly increased psychological distress, including prevalence of depression, anxiety, and loneliness (Brooks *et al*., 2020; Campion *et al*., 2020; Tyrer, 2020; Unützer *et al*., 2020).

Thus far, the vast majority of data available on psychological distress during COVID-19 are from high-income countries, while evidence from low- and middle-income countries (LMICs) has been scarce (Vindegaard and Benros, 2020; Xiong *et al*., 2020; Chen *et al*., 2021; Kola *et al*., 2021). The available evidence suggests that psychological distress during the pandemic has been high: for example, in a phone-based study in urban areas of Burkina Faso, Ethiopia, and Egypt, 28% of participants had symptoms of mild, moderate, or severe psychological distress (Workneh *et al*., 2021). LMIC populations may be particularly vulnerable to negative impacts of COVID-19-related policy measures due to higher rates of poverty, less comprehensive health and social care systems, and limited access to mental health services (Kola *et al*., 2021). Moreover, policy measures may further exacerbate systemic social inequities across sociocultural and socioeconomic groups (Gureje, 2020). Understanding levels of psychological distress in LMICs during COVID-19 is critical to informing policy responses to further waves of COVID-19, as well as future pandemics. It is also particularly salient given recent global health estimates that place mental illness, including depression and anxiety, as the leading cause of years of life lived with disability (Vigo *et al*., 2016).

Uganda is an important country in which to examine psychological distress during the COVID-19 pandemic because it implemented one of the most stringent lockdowns in the world. Beginning on 18 March 2020, several days before the first case of COVID-19 was confirmed in Uganda on 21 March 2020, the Ugandan government responded to the pandemic with a series of policy measures that included imposing a strict curfew; shutting down schools, businesses, and social gatherings; and severely limiting transportation (Hale *et al*., 2021). Uganda was one of several LMICs in which enforcement of COVID-19 policy measures entailed the use of violence by security officers (Katana *et al*., 2021). After a period of more relaxed measures, Uganda went into lockdown again starting in June 2021 in response to the second wave of COVID-19 cases. Ugandan schools did not fully reopen until January 2022, making Uganda the country with the longest school closures in the world (Blanshe and Dahir, 2022). Prior to the pandemic, the mental health care system in Uganda was already strained due to insufficient human resources and training, technical capacity in local government, and funding, circumstances which are similar to many other LMICs (Mugisha *et al*., 2016; Wakida *et al*., 2019; Sarikhani *et al*., 2021). While the Government of Uganda has made efforts to incorporate mental health care into the pandemic response, resource limitations have also led to some reductions in mental health service availability during the pandemic (Abbo *et al*., 2020). Furthermore, common sources of mental support in Uganda, particularly places of worship, were affected by COVID-19 lockdowns.

In this study, we measured levels of depression, anxiety, and psychological distress among adults in Uganda from December 2020 through April 2021, during the peak and near the end of the first COVID-19 wave. We then assessed associations of distress with experiences of policy measures implemented by the Government of Uganda in response to the pandemic and with sociodemographic factors.

## Methods

### Study setting

Uganda is situated in East Africa with a population of 46 million in 2020, nearly half of whom were under the age of 15 (The World Bank, 2020*a*, 2020*b*). In 2016, 41% of Ugandans lived on less than $1.90 per day (The World Bank, 2016). Mental health care is a component of the National Minimum Health Care Package. Mental health services are provided through the country's decentralized health care system, with outpatient and inpatient services available in the national referral psychiatric hospital in Kampala and at 13 regional referral hospitals (Ssebunnya *et al*., 2018). Resource and staffing levels are low, with are an estimated 1.42 psychiatric beds, 0.09 psychiatrists, 0.1 psychologists, 0.01 social workers, 0.2 psychiatric clinical officers, and 6.4 psychiatric nurses per 100 000 people (Ssebunnya *et al*., 2018). There is limited availability of community-level mental health care services, so poor and rural populations have few opportunities for accessing care (Ssebunnya *et al*., 2018). Pervasive stigmatization of mental illness in the general population and among health workers is a barrier to policy change (Mugisha *et al*., 2016). Previous research on mental health in Uganda has focused primarily on depression and post-traumatic stress disorder in the Northern region, where long-standing civil conflict led to years of violence (Mugisha *et al*., 2015). Some smaller studies of mental health in Uganda have estimated the prevalence of depression between 17% and 30% (Ovuga *et al*., 2005; Sweetland *et al*., 2019). Women and people living with HIV have also been identified as groups with a potentially elevated risk of depression (Ovuga *et al*., 2005; Sweetland *et al*., 2019).

The first case of COVID-19 in Uganda was confirmed on 22 March 2020, but it was not until August 2020 that Uganda identified community transmission of COVID-19. To date, Uganda has experienced three waves of COVID-19 cases: based on case counts, the first wave peaked in December 2020, the second wave peaked in June 2021, and the third wave peaked in January 2022 (Our World in Data, 2022; Johns Hopkins Coronavirus Resource Center, 2022). As of 28 February 2022, Uganda had recorded an estimated 163 231 cases of COVID-19 and 3585 deaths (World Health Organization, 2022).

The Ugandan government implemented one of the most stringent COVID-19 lockdowns in the world (Hale *et al*., 2021). Starting in March 2020, the government instituted a series of measures including a nationwide curfew, suspension of mass gatherings including communal prayers and weekly markets, school closures, suspension of public transport, restrictions on private vehicles, and border closures (Ministry of Health Uganda, 2020). Though initially instituted for 30 days, most lockdown measures were extended until September 2020. After that, the curfew was partially relaxed, though schools remained closed, gatherings were restricted to less than 200 people, and public transport operated at a limited capacity (Ministry of Health Uganda, 2020). In June 2021, a stricter lockdown was reintroduced in response to rising cases (Ministry of Health Uganda, 2020). Measures were relaxed again in early 2022, when the reopening of schools was shortly followed by the reopening of bars and restaurants (Al Jazeera, 2022; Blanshe and Dahir, 2022).

During the COVID-19 pandemic, Uganda followed World Health Organization guidance for incorporating mental health and psychosocial support (MHPSS) within the COVID-19 taskforce (Abbo *et al*., 2020). The MHPSS sub-pillar of the task force was formed at the beginning of the pandemic and included different individuals with expertise in mental health from a variety of organizations including the WHO, Ministry of Health (MoH), Butabika National Referral Mental Hospital, Makerere University, UNICEF, CDC, and local NGOs. Through these organizations, the MHPSS sub-pillar carried out several roles including cascading MHPSS trainings to community levels, and providing telephone services by Strong Minds Uganda, an international NGO. Mental health workers were deployed to quarantine and COVID-19 treatment centers around Kampala. However, the few existing regional mental health units were turned into COVID-19 treatment units, further restricting access to mental health services (Abbo *et al*., 2020). Despite advocacy efforts by the MHPSS sub-pillar of the COVID-19 taskforce, these units were not returned to mental health services until March 2022.

During the pandemic, the government implemented a variety of social protection measures to support vulnerable populations (Akina Mama Wa Afrika, 2020). Cash transfer programs for seniors and girls were modified to be delivered in a COVID-19-safe manner. Starting in April 2020, the government distributed food to vulnerable populations. Additional measures included debt relief measures and fee relief measures for mobile money transfers.

Still, the pandemic brought major disruptions to economic and social life in Uganda. Uganda's real gross domestic product growth decreased from 6.8% in 2019 to 2.9% in 2020 and an estimated 1.1 to 3.2 million people were driven into poverty (The World Bank, 2021). Social interactions were substantially reduced (Abbo *et al*., 2020; Ainamani *et al*., 2020). News outlets reported the use of force by security personnel against civilians during the lockdown, with at least 12 people killed during violent interactions with police (Ainamani *et al*., 2020; Akumu, 2020; The EastAfrican, 2020; Katana *et al*., 2021).

### Study design, sampling, and participants

Between December 2020 and April 2021, we conducted a monthly mobile phone survey with a random sample of 4066 adults aged 18 years or older in Uganda. The survey was conducted using interactive voice response (IVR), which uses voice recordings to ask participants a series of questions to which they reply using their touchpad. IVR is preferred over text messaging or app-based surveys in areas with low literacy, rural areas, and areas with low penetration of smartphones (Hensen *et al*., 2021). The survey was translated and available in English, Luganda, Luo, Ateso, and Runyakitara.

**Fig. 1.**
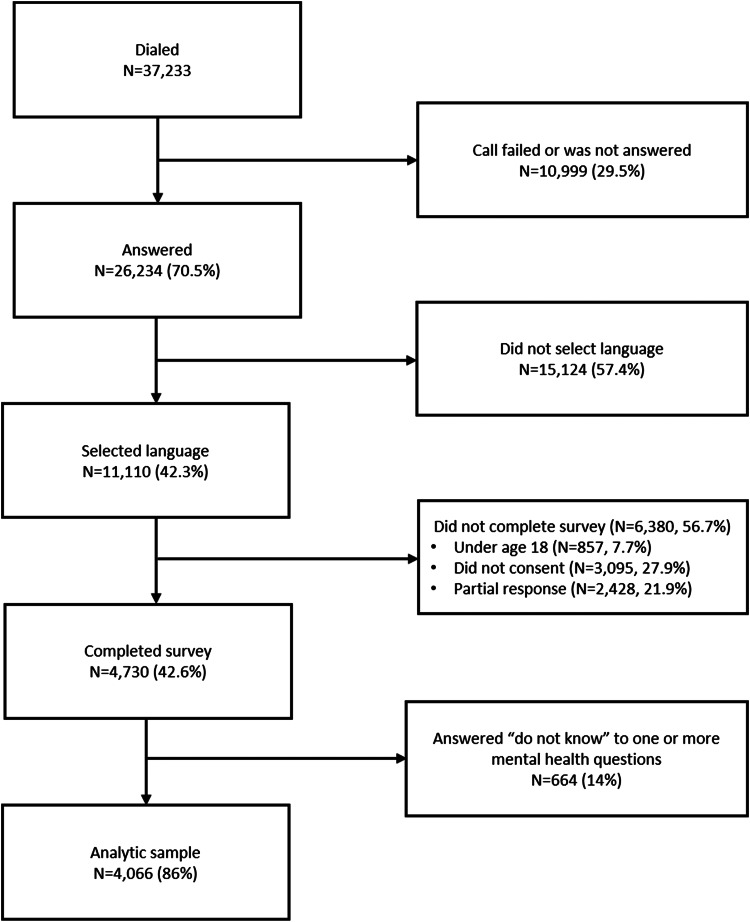
Study sample diagram.

The sampling frame was subscribers to the 3–2–1 service in Uganda, a popular free service on the Airtel network (the second largest communication network in Uganda) that provides subscribers with information on agriculture, financial services, and other topics via SMS and audio messages. About 500 000 individuals are fully registered subscribers, with about 38% in urban areas and 62% in rural areas. Each month between December 2020 and April 2021, the software automatically sampled phone numbers from the list of subscribers until a quota of 1000 subscribers completed the survey. Sampling was stratified by age, gender, and region of residence. Individuals who answered the phone call were asked for their language preference, then asked to provide consent by tapping a phone key. Participants who consented continued with the survey. This methodology allows for maximum sample size, as response rates for mobile phone surveys tend to be low: in our study, 70% of subscribers who were called answered the phone call, and of those that answered, 42% consented to start the survey (Fig. 1). These response rates are in line with recent literature on mobile phone surveys (Gibson *et al*., 2019). The sample size of 1000 participants per month was determined to be appropriate based on power calculations using the PHQ-9. After piloting, the survey was shortened to the PHQ-4 based on the high correlation between the PHQ-4 and PHQ-9 in the pilot sample.

Participation in the survey was free and participants earned a mobile ‘top-up’ of 2500 Ugandan shillings (approximately $0.68) upon completion. At the end of the call, participants were referred to an Uganda COVID-19 hotline for further information about COVID-19 and mental health, managed by the Ugandan Ministry of Health.

A pilot survey (*N* = 412) was conducted in November 2020 to examine question order, wording, and survey length. Following this, the survey was shortened and several questions were revised. Data from the pilot were not used in the analysis. There was a computer error in the platform used to administer the survey in February 2021: that month, most respondents were not given the choice of a language other than English. After verifying results were not sensitive to omitting these data, we included responses from February 2021 in our analysis.

### Study variables

Psychological distress was measured with the Patient Health Questionnaire (PHQ)-4, a brief self-report questionnaire to assess symptoms of depression and anxiety (Kroenke *et al*., 2009*a*). Originally validated in the U.S. (Kroenke *et al*., 2009*a*), the PHQ-4 has now additionally been validated in low-income countries (Barrera *et al*., 2020; Carroll *et al*., 2020). It has also been used to measure psychological distress during the pandemic in several countries via online and phone (voice) surveys (Schnell and Krampe, 2020; Zhang *et al*., 2020; Daly and Robinson, 2021; Workneh *et al*., 2021). While the PHQ-4 has not been validated in Uganda, both the PHQ-9 and PHQ-2 versions have been validated, with the PHQ-9 performing only slightly better than the PHQ-2 (Akena *et al*., 2013; Nakku *et al*., 2016).

The PHQ-4 consists of a 2-item depression scale (PHQ-2, which assesses ‘feeling down, depressed, and hopeless’ and ‘little interest or pleasure in doing things’) and a 2-item anxiety scale (General Anxiety Disorder (GAD)-2, which assesses ‘feeling nervous, anxious, or on edge’ and ‘not being able to stop worrying’). Participants indicate how often they were bothered by these problems over the last 2 weeks. The response set was modified for the Ugandan context to indicate days as opposed to stylized categories as follows: ‘not at all’ = 0 to 1 days, ‘several days’ = 2 to 6 days, ‘more than half the days’ = 7 to 11 days, and ‘nearly every day’ = 12 to 14 days, with points (0 to 3) assigned to each response set, respectively (Kroenke *et al*., 2009*b*). The total score is determined by adding points for each item and ranges from 0 to 12. Total scores are categorized as normal (0–2), mild (3–5), moderate (6–8), or severe (9–12). Scores of 3 or higher for the PHQ-2 items suggest possible depression, while scores of 3 or higher on the GAD-2 items suggest possible anxiety.

Explanatory variables measured experiences of COVID-19 and lockdown, and included the number of days in the past week respondent stayed at home the whole day, without going out at all and without receiving any visitors (0–1, 2–3, 4–5, or 6–7 days); needing to care for a child that would normally be in school or day care in the past 30 days (yes or no); inability to access needed medicine or medical care in the past 30 days (yes or no); change in income since March 2020 because of COVID-19 restrictions (gain, no change, small reduction, or large reduction); having to move elsewhere since March 2020 due to COVID-19 (yes or no); worry over themselves or someone close to them becoming infected with COVID-19 (not at all, some, or a lot); and worry over getting in trouble with police for violating lockdown measures (not at all, some, or a lot). Sociodemographic controls included age group, gender, urban/rural, marital status, language, highest education, household main source of income, number of children in household, any long-term physical or mental health conditions, and region. Questions and response options are included in online Supplementary Table S1.

### Statistical analysis

The study sample was defined as eligible participants who consented, completed the survey, and responded to the PHQ-4 questions. All analyses applied sampling weights calculated using a raking procedure to weight the sample to population characteristics (gender, age group, and region) derived from recent nationally representative household surveys (Lohr, 1999). We compared weighted and unweighted sample demographic characteristics to Ugandan population characteristics based on these household surveys.

The goal of our analysis was to determine whether COVID-19 experiences were correlated with PHQ-4 scores, and whether these associations remained after adjusting for sociodemographic characteristics of survey participants. We used a regression framework to perform this statistical adjustment. In our main analysis, associations between PHQ-4 score and explanatory variables were assessed using linear regression with robust standard errors. Our approach to model selection was to implement a range of alternative models and robustness checks to ensure estimates were not sensitive to model choice. We drew on prior empirical studies and theory from the social determinants of health literature to identify relevant explanatory variables (Lund *et al*., 2018). First, models were fit including sociodemographic characteristics and an indicator of survey round as the only covariates. Subsequently, we added COVID-19 explanatory variables to the model one at a time. We used separate regression models rather than one single model to avoid adjusting for variables on the causal pathway between the COVID-19 variable of interest and PHQ-4 score. We examined the *R*^2^ values for model fit. Because there were few missing values, we conducted a complete case analysis. However, results were very similar to using dummy variables for missing data. We summarized regression results using predicted values of PHQ-4 scores across COVID-19 related variables, adjusted for controls. The online Appendix shows the full regression output.

We conducted several sensitivity analyses. First, we excluded data from February 2021 due to the technical issue mentioned above. Second, to assess how non-response might impact on the composition of our sample, among those who completed demographic questions we compared participants who dropped out before reaching the PHQ-4 questions with those who completed all sections. Third, we examined the sensitivity of our findings to alternative outcome specification (PHQ-4 >= 6, as a binary measure of psychological distress using a logistic regression model); to alternative model specifications (generalized linear model using a negative binomial distribution); and to the omission of sampling weights. All analyses used Stata 16.

### Ethical approvals

This study received ethical approval at Rutgers School of Public Health (Pro2020001762), Makerere College of Health Sciences Higher Degrees, Research and Ethics Committee (#814), and the Uganda National Council of Science and Technology (HS1084ES).

### Role of the funding source

The funders had no role in study design; data collection, analysis, interpretation; writing; or the decision to submit the paper.

## Results

### Sample characteristics

Of 37 233 individuals contacted between December 2020 and April 2021, 26 234 (71%) answered the call and 11 110 (42%) selected a language. Of those who selected a language, 4730 (43%) started the survey. Of this group, 4066 (86%) completed all PHQ-4 questions and compose the final analytic sample. The study flow diagram for the sample is shown in Fig. 1.

Online Appendix Table S2 shows that the 1874 individuals who were over 18 and started but subsequently dropped out of the survey are similar in characteristics to the analytic sample, which strengthens our confidence that selection on observed characteristics is not a major source of bias.

Table 1 describes the characteristics of unweighted and weighted study samples, and compares the weighted sample to a nationally representative sample. While the unweighted sample was skewed to a younger and unmarried population, the weighted sample was similar to nationally representative population statistics. 59% of the weighted sample completed primary education or less, compared to 63% nationally, and 74% reported living in a village/rural area, which was the same nationally. About 47% of the weighted sample listed farming as the main source of income, compared to 43% nationally. Demographic characteristics were similar across survey months (Online Supplementary Table S3), except for the February 2021 round in which respondents were more likely to be male and have more education, which was the result of English being the only available language that round. Descriptive statistics showing the frequency with which participants experienced COVID-related events is shown in online Table S5 in the supplementary material.

**Table 1.** Unweighted and weighted sample characteristics, in comparison with population characteristics from recent household surveys (*N* = 4066)

	Unweighted sample	Weighted sample	Population estimates from household surveys
Characteristics	*N* (%)	% (95% CI)	% source (%)
Gender			
Female	1997 (49.1%)	52.0% (49.7–54.3%)	52^a^
Male	2069 (50.9%)	48.0% (45.7–50.3%)	48^a^
Age Group			
18 to 24 years	2536 (62.4%)	29.9% (28.3–31.5%)	30^a^
25 to 34 years	1060 (26.1%)	29.7% (27.9–31.6%)	30^a^
35 to 44 years	274 (6.7%)	18.7% (16.7–20.9%)	19^a^
45 years or older	196 (4.8%)	21.8% (19.3–24.6%)	22^a^
Marital Status			
Divorced, separated, or widowed	215 (5.4%)	7.0% (5.8–8.5%)	–
Have never married	1772 (44.4%)	30.9% (29.0–32.9%)	29^c^
Married or living with partner	2004 (50.2%)	62.0% (59.8–64.1%)	–
Highest Education			
No formal schooling	122 (3.0%)	3.2% (2.4–4.1%)	63^c^
Less than primary	617 (15.2%)	14.9% (13.4–16.7%)	
Completed primary	1720 (42.5%)	41.3% (39.0–43.6%)	
Completed secondary	1143 (28.2%)	26.5% (24.5–28.5%)	–
Completed tertiary	446 (11.0%)	14.1% (12.5–15.9%)	–
Household's Main Income Source before March 2020			
Business income	1375 (34.4%)	33.1% (39.9–35.2%)	–
Farming, livestock, fishing	1866 (46.7%)	46.5% (44.2–48.8%)	43^c^
Remittances	165 (4.1%)	4.1% (3.2–5.1%)	–
Wage-employment	435 (10.9%)	11.7% (10.2–13.2%)	25^c^
Other sources	159 (4.0%)	4.7% (3.7–5.8%)	–
Number of Children in Household			
No children	971 (24.0%)	18.3% (16.8–19.9%)	–
1 or 2 children	1787 (44.2%)	39.7% (37.5–42.0%)	–
3 or more children	1281 (31.7%)	42.0% (29.6–44.4%)	–
Long-term Health Condition			
No	2043 (50.8%)	51.9% (49.6–54.2%)	–
Yes	1982 (49.2%)	48.1% (45.8–50.4%)	–
Place of Residence			
City or town	1134 (28.2%)	25.9% (24.0–27.9%)	26^b^
Village	2892 (71.8%)	74.1% (72.1–76.0%)	74^b^
Region			
Central	1151 (28.3%)	27.6% (25.6–29.7%)	28^c^
Eastern	1739 (42.8%)	26.1% (24.4–27.8%)	26^c^
Northern	512 (12.6%)	20.8% (18.8–23.0%)	21^c^
Western	664 (16.3%)	25.5% (23.3–27.8%)	26^c^
Language of Survey			
Ateso	60 (1.5%)	0.9% (0.6–1.2%)	–
English	1070 (26.3%)	28.1% (26.1–30.4%)	–
Luganda	2036 (50.1%)	46.6% (44.3–48.9%)	–
Lugbara	12 (0.3%)	0.5% (0.2–1.0%)	–
Luo	184 (4.5%)	6.1% (5.0–7.4%)	–
Runyakitara	704 (17.3%)	17.8% (16.1–19.7%)	–

*Notes:* Table shows sample characteristics before and after sampling weights are applied, as well as population characteristics derived from recent household surveys in Uganda. ‘95% CI’ indicates the 95% confidence interval calculated around the weighted estimate. Population values are from the following sources: ^a^UN Population Division 2019; ^b^DHS 2016, for population aged 15–49; ^c^Uganda National Household Survey.

### Levels of psychological distress, anxiety, and depression

Figure 1 shows the percentage of respondents reporting psychological distress, anxiety, and depression, weighted to reflect the national population. An estimated 50.0% (95% CI 48.3% to 51.5%) of the population reported elevated PHQ-2 scores (indicating possible depression), while 44.8% (43.2% to 46.3%) of the sample reported elevated GAD-2 scores (indicating possible anxiety). An estimated 29.2% (27.8% to 30.6%) reported PHQ-4 scores from 6 to 8, indicating possible moderate levels of psychological distress, and 12.1% (11.1% to 13.1%) reported PHQ-4 scores 9 and above, indicating possible severe psychological distress. Online Supplementary Table S4 shows weighted estimates for composite and individual PHQ-4 items. Online Supplementary Fig. S2 shows distributions of PHQ-4, PHQ-2, and GAD-2 scores.

### Sociodemographic factors associated with psychological distress

**Fig. 2.**
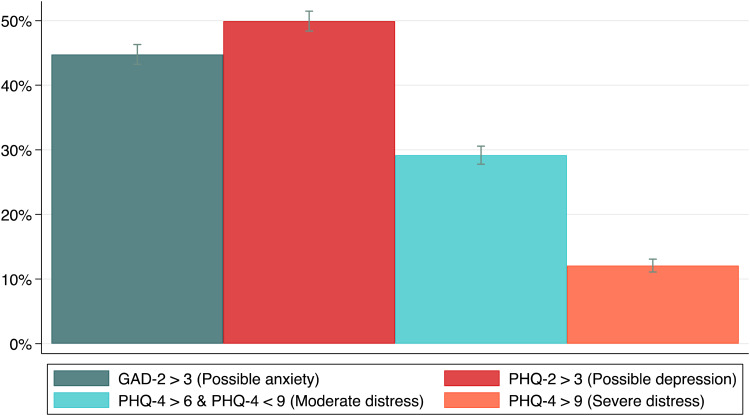
Percentage of respondents reporting elevated PHQ-4, PHQ-2, and GAD-2 scores in the study population. *Notes:* 95% confidence intervals are shown.

The average PHQ-4 total score (standard deviation) was 5.05 (2.9). Several sociodemographic characteristics were associated with higher psychological distress, as measured by total PHQ-4 score (Fig. 2). Conditional on all other characteristics, female gender was associated with a 0.44-point higher PHQ-4 score (95% CI 0.15 to 0.74), while having tertiary education compared with primary education was associated with a 1.0-point lower score (−1.46 to −0.56). Living in a household with three or more children compared with no children was associated with a 0.78-point higher score (0.44 to 1.13) and living in the Northern region was associated with a 0.74-point higher score (0.30 to 1.19) compared to the Central region.

### Predictors of psychological distress

**Fig. 3.**
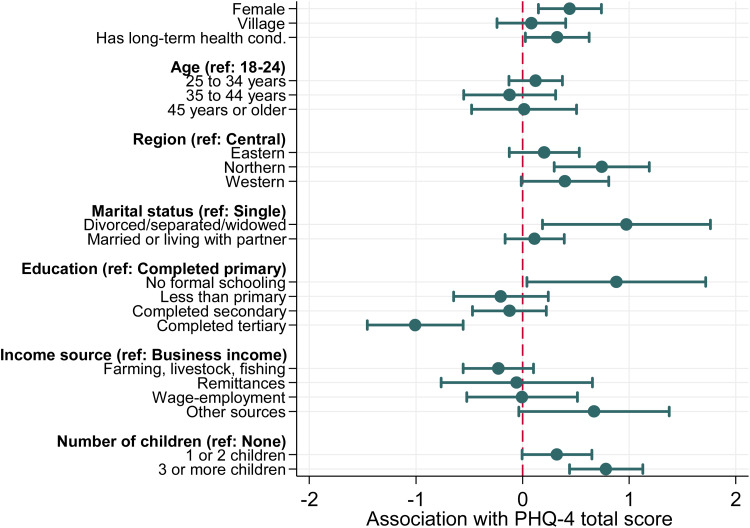
Association between demographic characteristics and PHQ-4 score. *Notes:* Figure shows output from linear regression of the Patient Health Questionnaire (PHQ)-4 score on respondents' demographic characteristics with robust standard errors. ‘Female’ is an indicator for female gender. ‘Village’ is an indicator for living in a village as opposed to an urban area. ‘Has long-term health cond.’ indicates that the respondent has a long-term health condition. For other variables shown, the reference category is included in the title (indicated by ‘ref’). Point estimates showing associations are linear regression coefficients, and that the dotted red line indicates a linear association of 0, the null hypothesis against which the estimated regression coefficient is tested against. 95% confidence intervals are shown.

**Fig. 4.**
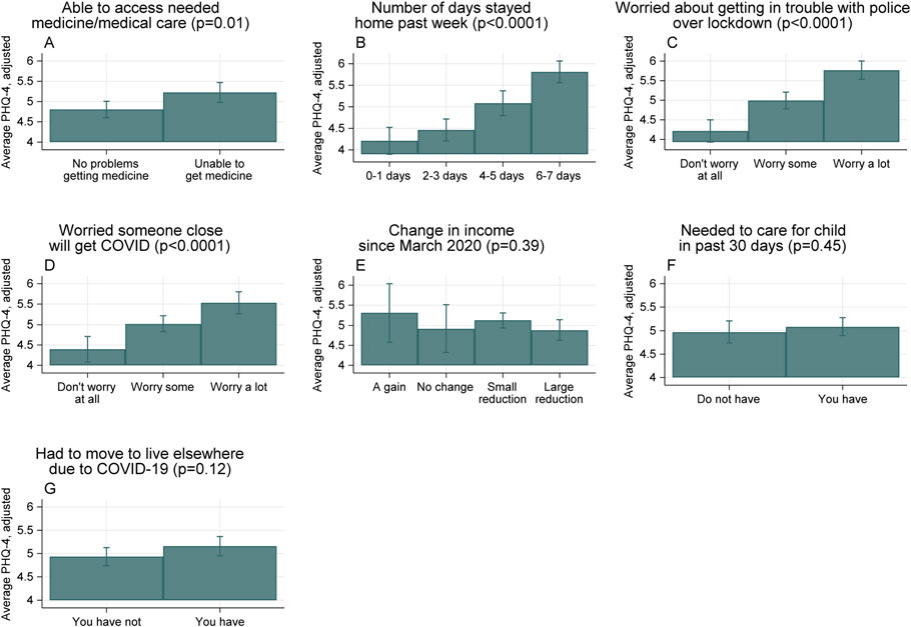
Predictors of psychological distress during COVID-19. *Notes:* Figure shows estimated average scores on the Patient Health Questionnaire (PHQ)-4 for subgroups of the study population, marginalized over respondent characteristics (survey round, age, urban *v.* rural location, gender, region, marital status, education level, income source, number of children in the household). Bars represent 95% confidence intervals. The *p* value shown after the title of each panel is from the *F* test comparing the coefficients for different levels of the relevant categorical variable.

Figure 3 shows associations between respondents' experiences during COVID-19 and their PHQ-4 scores, after adjusting for sociodemographic characteristics. Respondents who reported experiencing being unable to access needed medicines or medical care for themselves or household members had higher PHQ-4 scores compared to those not reporting these problems (5.2 *v.* 4.8, *p* value of difference = 0.009, Panel A). Social isolation related to COVID-19 was also significantly associated with greater psychological distress. Respondents who stayed home for more days in the past week had significantly higher PHQ-4 scores (Panel B). The mean PHQ-4 score for those who stayed home 6–7 days in the past week without going out or receiving visitors compared to 0–1 days was 5.8 *v.* 4.2, *p* value < 0.0001 (Fig. 4).

Respondents who reported worries related to COVID-19 had higher adjusted PHQ-4 scores (Panels C and D). Those who reported worrying a lot about getting in trouble with the police over lockdown procedures had higher PHQ-4 scores compared to those who did not report worrying (5.7 *v.* 4.2, *p* value < 0.0001). Respondents who reported worrying a lot about someone close to them getting COVID-19 had higher scores compared to those who did not report worrying about this (5.5 *v.* 4.4, *p* value < 0.0001). There was no clear pattern of psychological distress associated with income loss or gain (Panel E); however, the vast majority of individuals responded experiencing either a small or large income loss since March 2020 (93%) (Online Supplementary Table S5).

Needing to move to live elsewhere due to COVID-19 was associated with a slightly higher PHQ-4 score compared with not needing to move (5.2 *v.* 4.9, *p* value = 0.09 Panel G). The *R*^2^ from these linear models ranged from 5–10%. Online Supplementary Table S6 shows the full regression output. The main conclusions were robust to sensitivity analyses including alternative model specifications (Online Supplementary Table S7).

## Discussion

The aims of this study were to estimate the levels of anxiety, depression, and psychological distress among adults in Uganda during the COVID-19 pandemic, and examine associations between psychological distress and experiences during the pandemic. We found high levels of psychological distress among Ugandan adults in the year following the start of the pandemic: 41% of adults reported elevated PHQ-4 scores, indicating either moderate (29%) or severe (12%) levels of psychological distress. These estimates are near the upper end of the range of results from a global systematic review of the effects of COVID-19 on psychological outcomes, where the prevalence of anxiety ranged from 6% to 51% and depression ranged from 15% to 48% across eight countries, though the majority of countries included in the review were upper-middle or high income (Xiong *et al*., 2020). Our estimates are also on the high end of estimates from the few studies conducted in low-income countries, where the prevalence of depression or mental health symptoms ranged from 7% to 52% (Cénat *et al*., 2021; Langsi *et al*., 2021; Mamun *et al*., 2021; Workneh *et al*., 2021; Logie *et al*., 2022). While we do not have data collected from before the start of the pandemic in Uganda, our estimates indicate higher rates of psychological distress compared to a 2018 study, which found that 31% of individuals reported moderate or severe mental distress in Uganda, as measured by the Kessler-6 scale (Sweetland *et al*., 2019). Other previous studies in Uganda found the prevalence of depression to be between 17% and 28%, although differences in sampling and measurement tools make it difficult to directly compare estimates (Ovuga *et al*., 2005; Logie *et al*., 2022).

We found that the sociodemographic groups most at risk of experiencing psychological distress during COVID-19 included women, those with lower levels of education, and those living in households with children. These findings are consistent with the results of COVID-19 mental health studies from other countries (Xiong *et al*., 2020; Langsi *et al*., 2021; Mamun *et al*., 2021; Mei *et al*., 2021). The magnitudes of these relationships are substantial and point to important sociodemographic disparities in psychological distress. For example, the difference in PHQ-4 scores between the highest (tertiary education) and lowest (no formal education) education groups is two PHQ-4 score units, equivalent to adding two additional symptoms (e.g. feeling nervous, anxious, or on edge; not being able to stop or control worrying), or increasing the frequency of symptoms by two categories (such as moving from experiencing one of these symptoms 0–1 days to 7–11 days). The association between psychological distress and the number of children living in the household suggests the pandemic may have disproportionately affected families with children. We also found higher rates of psychological distress in the Northern region. Geographic differences in outcomes may be driven by longstanding mental trauma effects of the violent conflict in the Northern region and internal displacement, as well as the recent increase of refugees from South Sudan to northern Uganda (Mugisha *et al*., 2015).

We also found strong associations between psychological distress and experiences of COVID-19 policy-related variables. We found a pronounced gradient in distress across the number of days spent at home in isolation in the past week, which is consistent with research on social withdrawal (Rooksby *et al*., 2020; Holt-Lunstad, 2021). Social isolation is particularly important in the cultural context of Uganda, where social cohesion and support are crucial in times of adversity, and participation in religious gatherings is an important coping strategy for many people (Ainamani *et al*., 2020). We also found a strong gradient in psychological distress across levels of worry over getting in trouble with the police over violating lockdown measures. The United Nations human rights office found more than a dozen countries employed exceptional emergency measures to enforce curfews and social distancing that included the use of excessive and deadly force by police (Farge, 2020), and the Ugandan media published several articles profiling police brutality (Akumu, 2020; The EastAfrican, 2020). Yet, there has been little research to date on the impacts of police brutality and enforced isolation on population mental health in LMICs (Rahman *et al*., 2021). Our results suggest violent enforcement has severe psychological consequences for the general population.

The Ugandan COVID-19 National Task Force took fast action to minimize the transmission of COVID-19, and the recent surge in cases has necessitated further lockdown measures in Uganda. While social protection measures to support vulnerable populations were implemented, these were insufficient to meet needs (Akina Mama Wa Afrika, 2020). Our findings suggest that, when lockdowns are needed, countries must consider how to mitigate their mental health consequences, including improving policing quality to reduce the violence of law enforcement against citizens and building strong social safety nets to ensure access to medical and social services for vulnerable populations. These findings underscore recommendations for countries to embed policies to protect human rights and social and economic freedom in their pandemic responses (Rahman *et al*., 2021). Moreover, COVID-19 is not the first nor will it be the last pandemic and important lessons can be learned from COVID-19 and previous public health emergencies, such as HIV and Ebola, regarding their impact on population mental health (Mohammed *et al*., 2015; Kamara *et al*., 2017; Cénat *et al*., 2020). Policymakers must recognize the psychological impact of pandemics, epidemics, and disease outbreaks on the general population, not just infected individuals, particularly when lockdowns are implemented, and focus efforts on the larger social environment (Chew *et al*., 2020). Psychosocial support at the population level is critical for addressing fear and stigma, increasing community empowerment and collaboration, and facilitating access to services (Garoff, 2015; PAHO/WHO, 2016; Chew *et al*., 2020).

In addition, our findings suggest urgent action is needed to address mental health in Uganda and other LMICs. COVID-19 has likely exacerbated the large and increasing burden of mental health disorders in LMICs, where lack of mental health resources and less access to evidence-based interventions had already created a significant treatment gap (Vigo *et al*., 2016). In Uganda, as in many LMICs, there are multiple challenges to increasing access to mental health services, including chronic underfunding, fragmented service delivery models, lack of trained health providers, and stigma (Mugisha *et al*., 2016; Abbo *et al*., 2020). There is a need for increased funding to scale up mental health services, reduce fragmentation, and integrate mental health services into primary care (Patel *et al*., 2013; Arenliu *et al*., 2020; Jaguga and Kwobah, 2020; Kola *et al*., 2021; Molebatsi *et al*., 2021; Small and Blanc, 2021). The pandemic may present a window of opportunity to address these long-standing mental health treatment gaps in LMICs.

This study has several limitations. First, while our study did not aim to evaluate changes in mental health before and after the pandemic, such baseline data would be helpful in better understanding how the prevalence of psychological distress has changed with the pandemic. However, observed associations between psychological distress and pandemic-related variables suggest changes brought about by the pandemic have been important for mental health. Second, while mobile phone surveys have important advantages including reach, safety, confidentiality, and cost-effectiveness that make them ideal for use during the pandemic (Hensen *et al*., 2021), it is not possible to reach a fully representative sample of the population. About 70% of the total population in Uganda own mobile phones; males and those living in urban areas are more likely to own phones than their counterparts (NITA Uganda, 2018). Survey weighting, as this study uses, can partially but not fully address this as the population with phones may differ on unobservable characteristics from the population without phones or those who do not answer the phone or complete the survey. Third, while the PHQ-4 has been used in online and phone (voice) surveys, it has not to our knowledge been used in other studies using IVR phone surveys. Fourth, while our analysis pools data gathered from participants at various stages of the pandemic (as measured by differences in the levels of COVID-19 prevalence, mortality rates, and implementation of lockdown policies), analysis stratified by month showed similar results. Finally, we estimate associations in our data. Future research could compare mental health outcomes across countries and regions with varying lockdown measures using quasi-experimental methods to assess the causal impact of policy responses.

## Conclusions

Overall, the high rates of psychological distress observed in this study reflect the impact of the indirect effects of COVID-19 in Uganda, where infection rates remained low during the study period. The situation may have worsened during later waves of COVID-19, driven by the experience of illness, grief, and prolonged lockdown measures. These effects may be long-lasting and future research should assess the long-term consequences of the COVID-19 pandemic and policy response on mental health in LMICs. Our findings bring into focus the importance of providing health and social care protections as part of COVID-19 response policies, and underscore the urgent need to increase mental health support for people experiencing psychological distress (Ainamani *et al*., 2020; Ghebreyesus, 2020; Rahman *et al*., 2021). Finally, results highlight the urgent need to scale-up global investments in building resilient health systems that can withstand health crises beyond COVID-19.

## Supplementary material

For supplementary material accompanying this paper visit https://doi.org/10.1017/gmh.2022.28. click here to view supplementary material
