# Supplementary material for: Levels of depression, anxiety, and psychological distress among Ugandan adults during the first wave of the COVID-19 pandemic: cross-sectional evidence from a mobile phone-based population survey
Source: Glob Ment Health (Camb). 2022 Jun 30;9:274–84. doi: 10.1017/gmh.2022.28 (PMC9807010; doi:10.1017/gmh.2022.28)
Supplement: Supplementary file 1 [file S2054425122000280sup001.docx]

**Supplementary Appendix to:**

**Experiences of COVID-19 policy measures and mental health in Uganda: cross-sectional evidence from a mobile phone-based population survey**

**Figure S1: Histogram of sampling weights**

**Figure S2: Weighted distributions of PHQ-4, PHQ-2, and GAD-2 scores in the study population**

Notes: Figure shows the distribution of PHQ-4, PHQ-2, and GAD-2 scores in the study population. Histograms reflect sample weighting. The distribution of PHQ-4 total scores is shown in blue, with darker shades representing moderate and severe distress, respectively. The distribution of PHQ-2 scores is shown in green, with the darker shade indicating elevated scores. The distribution of GAD-2 scores is shown in red, with the darker shade indicating elevated scores.

**Table S1: Survey questions and response options**

| **Questions** | **Response options** |
| --- | --- |
| How old are you? | If you are under 18 years, press 1 If you are between 18 to 24 years, press 2 If you are between 25 to 34 years, press 3 If you are between 35 to 44 years, press 4  If you are 45 years or older, press 5 |
| Do you live in an urban area (like a city or town) or a rural area (like a village)? | If you live in a City or town, press 1 If you live in the Village, press 2 If you do not know, press 9 |
| What is your gender? | If you are Male, press 1 If you are Female, press 2 |
| What is your marital status? | If you have never married, press 1 If you are married or living with your partner, press 2 If you are Divorced/separated/widowed, press 3 If you do not know, press 9 |
| *Please think about the last 14 days, from two weeks ago until today. We would like to know how often during this 14-day period you were bothered by the following problems. There are four possible answer choices for each question – “0-1 days”, “2-6 days”, “7-11 days” or “12-14 days”.* | |
| In the last 14 days, how many days have you had little interest or enjoyment in doing things? | If it was between 0-1 days, press 1 If it was between 2-6 days, press 2 If it was between 7-11 days, press 3 If it was between 12-14 days, press 4 If you don't know, press 9 |
| In the last 14 days, how many days have you been feeling down, depressed or without hope? | If it was between 0-1 days, press 1 If it was between 2-6 days, press 2 If it was between 7-11 days, press 3 If it was between 12-14 days, press 4 If you do not know press 9 |
| In the last 14 days, how many days have you been feeling nervous, anxious, or on edge? | If it was between 0-1 days, press 1 If it was between 2-6 days, press 2 If it was between 7-11 days, press 3 If it was between 12-14 days, press 4 If you do not know, press 9 |
| In the last 14 days, how many days have you been unable to stop or control worrying? | If it was between 0-1 days, press 1 If it was between 2-6 days, press 2 If it was between 7-11 days, press 3 If it was between 12-14 days, press 4 If you do not know, press 9 |
| Do you have any long-term health issues such as diabetes, high blood pressure, heart disease, HIV, mental illness, or other? | If you do not have, press 1 If you have, press 2 If you don’t know, press 9 |
| *Now we would like to ask you about how COVID-19 has affected you.* | |
| In the past 7 days, how many days have you stayed at home the whole day, without going out at all and without receiving any visitors? | If it was between 0-1 days, press 1 If it was between 2-3 days, press 2 If it was between 4-5 days, press 3 If it was between 6-7 days, press 4 If you do not know, press 9 |
| In the past 30 days, have you needed to care for a child who would normally be in school or day care? | If you have, press 1 If you have not, press 2 If you do not know, press 9 |
| In the past 30 days, have you or any other member of your household been unable to obtain medicine or medical care or treatment that was needed? | If you have been unable to obtain medicine or medical care, press 1 If you have not had any problems obtaining medicine or medical care, press 2 If no one in your household has needed any medicine or medical care, press 3 If you do not know, press 9 |
| *For the next questions, think about the past months since March 20, 2020, the day the schools were closed.* | |
| Since March 20^th^, 2020, have you experienced a change in income because of COVID-19 or related restrictions? | If you have experienced a large reduction in income, press 1 If you have experienced a small reduction in income, press 2 If you have not experienced any change in income, press 3 If you have experienced a gain in income, press 4 If you do not know, press 9 |
| Since March 20^th^, 2020, have you had to go and live somewhere else because of COVID-19 or related restrictions? | If you have, press 1 If you have not, press 2 If you do not know, press 9 |
| At the moment, how much do you worry about you or someone close to you getting COVID-19? | If you don’t worry at all, press 1 If you worry some about it, press 2 If you worry a lot about it, press 3 If you do not know, press 9 |
| At the moment, how much do you worry about getting into trouble with the police or LDU for violating lockdown measures related to coronavirus, such as the curfew? | If you don’t worry at all, press 1 If you worry some about it, press 2 If you worry a lot about it, press 3 If you do not know, press 9 |
| What is the highest level of education you ever completed? | If it is Less than primary, press 1 If you completed primary, press 2 If you completed secondary, press 3 If you completed tertiary, press 4 If you have had no formal schooling, press 5 If you do not know, press 9 |
| What was your household’s main source of income before March 20th 2020, that is before the schools closed due to COVID-19? | If it is farming, livestock, fishing, press 1 If it is Business income (This includes income from business such as any type of non-farm trade, shops, saloons etc) , press 2 If it is from wage-employment, press 3 If it is from Remittances, press 4 If it is from other sources (such as: government assistance; assistance from NGO or charitable organization; pension; savings; income from property; investment; other), press 5 If you do not know, press 9 |
| How many children (under 18) do you live with under the same roof? | If there are no children, press 1 If they are 1 or 2 children, press 2 If they are 3 or more children, press 3 If you do not know, press 9 |
| What region are you currently in? | If it is the Central region, press 1 If it is the Eastern region, press 2 If it is the East central region, press 3 If it is the Northern region, press 4 If it is the north Eastern/ Karamoja region, press 5 If it is the North west / west Nile region, press 6 If it is the Western region, presss 7 If it is the Southwestern region, press 8 |

**Table S2: Demographic characteristics of respondents who dropped out of the survey**

|  | **Drop-out sample** | |  | **Mental health sample** | |
| --- | --- | --- | --- | --- | --- |
| **Variable** | **No.** | **%** |  | **No.** | **%** |
| **Age of respondent** |  |  |  |  |  |
| 18 to 24 years | 1064 | 56.9 |  | 2232 | 62.8 |
| 25 to 34 years | 421 | 22.5 |  | 922 | 25.9 |
| 35 to 44 years | 189 | 10.1 |  | 235 | 6.6 |
| 45 years or older | 196 | 10.5 |  | 164 | 4.6 |
| Total | 1870 | 100 |  | 3553 | 100 |
|  |  |  |  |  |  |
|  |  |  |  |  |  |
| **Place of residence** |  |  |  |  |  |
| City or town | 441 | 28 |  | 990 | 28.2 |
| Village | 1133 | 72 |  | 2524 | 71.8 |
| Total | 1574 | 100 |  | 3514 | 100 |
|  |  |  |  |  |  |
| **Gender of respondent** |  |  |  |  |  |
| Female | 704 | 44.6 |  | 1750 | 49.2 |
| Male | 873 | 55.4 |  | 1804 | 50.8 |
| Total | 1577 | 100 |  | 3554 | 100 |
|  |  |  |  |  |  |
| **Marital status of respondent** |  |  |  |  |  |
| Divorced/separated/widowed | 96 | 6.7 |  | 193 | 5.5 |
| Have never married | 613 | 43 |  | 1544 | 44.3 |
| Married or living with partner | 718 | 50.3 |  | 1745 | 50.1 |
| Total | 1427 | 100 |  | 3482 | 100 |

Notes: Columns 1 and 2 of the table show the characteristics of respondents in rounds 2-4 of the survey who completed demographic questions (“drop-out sample”) which were asked at the beginning of the survey but who did not complete any mental health questions because they dropped out. Columns 3 and 4 show the same characteristics for those who remained in the survey and completed all mental health questions.

**Table S3: Unweighted sample demographics by month**

|  | 12/2020 | 01/2021 | 02/2021 | 03/2021 | 04/2021 |
| --- | --- | --- | --- | --- | --- |
|  | N=495 | N=842 | N=890 | N=934 | N=905 |
| *Age Group* |  |  |  |  |  |
| 18 to 24 years | 279 (56.4%) | 506 (60.1%) | 555 (62.4%) | 573 (61.3%) | 623 (68.8%) |
| 25 to 34 years | 144 (29.1%) | 206 (24.5%) | 238 (26.7%) | 253 (27.1%) | 219 (24.2%) |
| 35 to 44 years | 39 (7.9%) | 70 (8.3%) | 60 (6.7%) | 57 (6.1%) | 48 (5.3%) |
| 45 years or older | 33 (6.7%) | 60 (7.1%) | 37 (4.2%) | 51 (5.5%) | 15 (1.7%) |
| *Place of Residence* |  |  |  |  |  |
| City or town | 125 (25.4%) | 246 (29.5%) | 224 (25.5%) | 260 (28.1%) | 279 (31.1%) |
| Village | 367 (74.6%) | 588 (70.5%) | 654 (74.5%) | 664 (71.9%) | 619 (68.9%) |
| *Gender* |  |  |  |  |  |
| Female | 244 (49.3%) | 272 (32.3%) | 563 (63.3%) | 466 (49.9%) | 452 (49.9%) |
| Male | 251 (50.7%) | 570 (67.7%) | 327 (36.7%) | 468 (50.1%) | 453 (50.1%) |
| *Marital Status* |  |  |  |  |  |
| Divorced/separated/widowed | 21 (4.3%) | 39 (4.7%) | 45 (5.2%) | 58 (6.3%) | 52 (5.9%) |
| Have never married | 197 (40.3%) | 423 (51.3%) | 377 (43.3%) | 373 (40.5%) | 402 (45.3%) |
| Married or living with partner | 271 (55.4%) | 362 (43.9%) | 449 (51.5%) | 489 (53.2%) | 433 (48.8%) |
| *Language* |  |  |  |  |  |
| Ateso | 9 (1.8%) | 1 (0.1%) | 13 (1.5%) | 5 (0.5%) | 32 (3.5%) |
| English | 44 (8.9%) | 762 (90.5%) | 69 (7.8%) | 94 (10.1%) | 101 (11.2%) |
| Luganda | 293 (59.2%) | 59 (7.0%) | 644 (72.4%) | 507 (54.3%) | 533 (58.9%) |
| Lugbara | 3 (0.6%) | 0 (0.0%) | 2 (0.2%) | 5 (0.5%) | 2 (0.2%) |
| Luo | 31 (6.3%) | 2 (0.2%) | 36 (4.0%) | 70 (7.5%) | 45 (5.0%) |
| Runyakitara | 115 (23.2%) | 18 (2.1%) | 126 (14.2%) | 253 (27.1%) | 192 (21.2%) |
| *Highest Education* |  |  |  |  |  |
| No formal schooling | 18 (3.6%) | 19 (2.3%) | 24 (2.7%) | 37 (4.0%) | 24 (2.7%) |
| Less than primary | 84 (17.0%) | 85 (10.2%) | 130 (14.6%) | 170 (18.2%) | 148 (16.4%) |
| Completed primary | 211 (42.7%) | 292 (35.0%) | 454 (51.1%) | 381 (40.9%) | 382 (42.4%) |
| Completed secondary | 132 (26.7%) | 314 (37.6%) | 197 (22.2%) | 251 (26.9%) | 249 (27.7%) |
| Completed tertiary | 49 (9.9%) | 124 (14.9%) | 83 (9.3%) | 93 (10.0%) | 97 (10.8%) |
| *Household's Main Income Source before March 2020* |  |  |  |  |  |
| Business income | 162 (33.5%) | 332 (40.4%) | 295 (33.6%) | 290 (31.5%) | 296 (33.0%) |
| Farming, livestock, fishing | 230 (47.6%) | 330 (40.1%) | 429 (48.9%) | 452 (49.0%) | 425 (47.4%) |
| Remittances | 21 (4.3%) | 18 (2.2%) | 31 (3.5%) | 50 (5.4%) | 45 (5.0%) |
| Wage-employment | 51 (10.6%) | 101 (12.3%) | 88 (10.0%) | 100 (10.8%) | 95 (10.6%) |
| Other sources | 19 (3.9%) | 41 (5.0%) | 34 (3.9%) | 30 (3.3%) | 35 (3.9%) |
| *Region* |  |  |  |  |  |
| Central | 132 (26.7%) | 256 (30.4%) | 284 (31.9%) | 216 (23.1%) | 263 (29.1%) |
| Eastern | 222 (44.8%) | 318 (37.8%) | 404 (45.4%) | 406 (43.5%) | 389 (43.0%) |
| Northern | 64 (12.9%) | 82 (9.7%) | 95 (10.7%) | 153 (16.4%) | 118 (13.0%) |
| Western | 77 (15.6%) | 186 (22.1%) | 107 (12.0%) | 159 (17.0%) | 135 (14.9%) |
| *Number of Children in Household* |  |  |  |  |  |
| No children | 84 (17.1%) | 191 (23.0%) | 218 (24.5%) | 235 (25.2%) | 243 (27.0%) |
| 1 or 2 children | 232 (47.2%) | 351 (42.3%) | 388 (43.7%) | 405 (43.5%) | 411 (45.7%) |
| 3 or more children | 176 (35.8%) | 287 (34.6%) | 282 (31.8%) | 291 (31.3%) | 245 (27.3%) |
| *Long-term Health Condition* |  |  |  |  |  |
| Do not have | 220 (45.9%) | 615 (73.2%) | 348 (39.3%) | 455 (49.2%) | 405 (45.2%) |
| You have | 259 (54.1%) | 225 (26.8%) | 537 (60.7%) | 469 (50.8%) | 492 (54.8%) |

**Table S4: PHQ-4 item responses and mental health outcomes in the study population (N=4,066)**

| **Outcome** | **% of respondents**  **(95% Confidence Interval)** |
| --- | --- |
| **Composite outcomes** |  |
| PHQ-2 >= 3 | 50.0%  (48.3% - 51.5%) |
| GAD-2 >= 3 | 44.8%  (43.2% - 46.3%) |
| PHQ-4 > 6 & PHQ-4 <=8 (“Moderate distress”) | 29.2%  (27.8% - 30.6%) |
| PHQ-4 > 8 (“Severe distress”) | 12.1%  (11.1% - 13.1%) |
| PHQ-4 total score (Mean (SD)) | 5.05 (2.9) |
| **Individual questions** |  |
| In the past 14 days, how many days have you had little interest or enjoyment in doing things? |  |
| 0-1 days | 20.0%  (18.2% - 21.8%) |
| 2-6 days | 38.6%  (36.4% - 40.9%) |
| 7-11 days | 23.4%  (21.5% - 25.4%) |
| 12-14 days | 18.0%  (16.2% - 20.0%) |
| In the past 14 days, how many days have you been feeling down, depressed, or without hope? |  |
| 0-1 days | 26.4%  (24.4% - 28.4%) |
| 2-6 days | 35.4%  (33.2% - 37.7%) |
| 7-11 days | 21.2%  (19.3% - 23.2%) |
| 12-14 days | 17.0%  (15.3% - 18.9%) |
| In the past 14 days, how many days have you been feeling nervous, anxious, or on edge? |  |
| 0-1 days | 28.9%  (26.8% - 31.1%) |
| 2-6 days | 34.8%  (32.6% - 37.0%) |
| 7-11 days | 20.8%  (19.0% - 22.7%) |
| 12-14 days | 15.6%  (13.9% - 17.4%) |
| In the past 14 days, how many days have you been unable to stop or control worrying? |  |
| 0-1 days | 34.4%  (32.2% - 36.7%) |
| 2-6 days | 32.9%  (30.8% - 35.0%) |
| 7-11 days | 17.1%  (15.4% - 18.9%) |
| 12-14 days | 15.6%  (14.0% - 17.5%) |

Notes: This table shows the estimated prevalence of mental health outcomes and of specific responses to mental health questions in the study population. Estimates reflect sample weighting.

**Table S5: Distribution of responses to survey questions about COVID-19 experiences (weighted)**

| **Outcome** | **N** | **%^a^** |
| --- | --- | --- |
| In the past 7 days, how many days have you stayed at home the whole day, without going out at all and without receiving any visitors? | 3,939 |  |
| No problems getting medicine |  | 46 |
| Unable to get medicine |  | 31 |
| Medicine not required |  | 23 |
| In the past 7 days, how many days have you stayed at home the whole day, without going out at all and without receiving any visitors? | 3,792 |  |
| 0-1 days |  | 19 |
| 2-3 days |  | 25 |
| 4-5 days |  | 22 |
| 6-7 days |  | 35 |
| At the moment, how much do you worry about getting into trouble with the police or LDU for violating lockdown measures related to coronavirus, such as the curfew? | 3,993 |  |
| Don’t worry at all |  | 25 |
| Worry some |  | 43 |
| Worry a lot |  | 32 |
| At the moment, how much do you worry about you or someone close to you getting COVID-19? | 3,998 |  |
| Don’t worry at all |  | 19 |
| Worry some |  | 52 |
| Worry a lot |  | 29 |
| Since March 20^th^, 2020, have you experienced a change in income because of COVID-19 or related restrictions? | 4,028 |  |
| A gain |  | 4 |
| No change |  | 3 |
| Small reduction |  | 60 |
| Large reduction |  | 33 |
| In the past 30 days, have you needed to care for a child who would normally be in school or day care? | 3,738 |  |
| Have not needed to |  | 43 |
| Have needed to |  | 57 |
| Since March 20^th^, 2020, have you had to go and live somewhere else because of COVID-19 or related restrictions? | 4,047 |  |
| Have not |  | 54 |
| Have |  | 46 |

Notes: ^a^Weighted with sampling weights.

**Table S6: Linear regression results of COVID-19 related variables on PHQ-4 total score, adjusted for sociodemographics**

| **Model** | **Unable to get med** | **Number days stayed home** | **Worried about police** | **Worried about COVID** | **Change in income** | **Childcare** | **Relocation** |
| --- | --- | --- | --- | --- | --- | --- | --- |
| **Able to get needed medicine/medical care** |  |  |  |  |  |  |  |
| No problems getting medicine (Ref) | 0 |  |  |  |  |  |  |
|  | (.) |  |  |  |  |  |  |
| Unable to get medicine | 0.420** |  |  |  |  |  |  |
|  | (0.162) |  |  |  |  |  |  |
| Medicine not required | 0.447* |  |  |  |  |  |  |
|  | (0.205) |  |  |  |  |  |  |
| **Number of days stayed home** |  |  |  |  |  |  |  |
| 0-1 days (Ref) |  | 0 |  |  |  |  |  |
|  |  | (.) |  |  |  |  |  |
| 2-3 days |  | 0.252 |  |  |  |  |  |
|  |  | (0.204) |  |  |  |  |  |
| 4-5 days |  | 0.873*** |  |  |  |  |  |
|  |  | (0.215) |  |  |  |  |  |
| 6-7 days |  | 1.600*** |  |  |  |  |  |
|  |  | (0.206) |  |  |  |  |  |
| **Worried about getting in trouble with police over lockdown** |  |  |  |  |  |  |  |
| Don't worry at all (Ref) |  |  | 0 |  |  |  |  |
|  |  |  | (.) |  |  |  |  |
| Worry some |  |  | 0.778*** |  |  |  |  |
|  |  |  | (0.181) |  |  |  |  |
| Worry a lot |  |  | 1.552*** |  |  |  |  |
|  |  |  | (0.189) |  |  |  |  |
| **Worried about someone close getting infected with COVID-19** |  |  |  |  |  |  |  |
| Don't worry at all (Ref) |  |  |  | 0 |  |  |  |
|  |  |  |  | (.) |  |  |  |
| Worry some |  |  |  | 0.623*** |  |  |  |
|  |  |  |  | (0.186) |  |  |  |
| Worry a lot |  |  |  | 1.133*** |  |  |  |
|  |  |  |  | (0.214) |  |  |  |
| **Change in income since March 2020** |  |  |  |  |  |  |  |
| A gain (Ref) |  |  |  |  | 0 |  |  |
|  |  |  |  |  | (.) |  |  |
| No change |  |  |  |  | -0.389 |  |  |
|  |  |  |  |  | (0.475) |  |  |
| Small reduction |  |  |  |  | -0.186 |  |  |
|  |  |  |  |  | (0.390) |  |  |
| Large Reduction |  |  |  |  | -0.426 |  |  |
|  |  |  |  |  | (0.394) |  |  |
| **Needed to care for a child who would normally be in school** |  |  |  |  |  |  |  |
| Do not have (Ref) |  |  |  |  |  | 0 |  |
|  |  |  |  |  |  | (.) |  |
| You have |  |  |  |  |  | 0.115 |  |
|  |  |  |  |  |  | (0.151) |  |
| **Needed to move elsewhere due to COVID-19** |  |  |  |  |  |  |  |
| You have not (Ref) |  |  |  |  |  |  | 0 |
|  |  |  |  |  |  |  | (.) |
| You have |  |  |  |  |  |  | 0.224 |
|  |  |  |  |  |  |  | (0.145) |
| **Survey round (ref. = round 1)** |  |  |  |  |  |  |  |
| round=2 | 0.381 | 0.361 | 0.443 | 0.339 | 0.367 | 0.376 | 0.332 |
|  | (0.273) | (0.260) | (0.271) | (0.272) | (0.273) | (0.280) | (0.269) |
| round=3 | -0.0819 | 0.0281 | 0.0132 | 0.0246 | -0.0443 | -0.0102 | -0.0234 |
|  | (0.259) | (0.252) | (0.258) | (0.261) | (0.256) | (0.265) | (0.256) |
| round=4 | -0.0324 | -0.0148 | 0.0989 | 0.0623 | -0.0440 | -0.00828 | -0.0594 |
|  | (0.252) | (0.244) | (0.251) | (0.253) | (0.250) | (0.257) | (0.250) |
| round=5 | 0.154 | 0.0845 | 0.110 | 0.156 | 0.148 | 0.128 | 0.117 |
|  | (0.254) | (0.248) | (0.249) | (0.252) | (0.249) | (0.257) | (0.249) |
| **Age group (ref. = 18-24 years)** |  |  |  |  |  |  |  |
| 25 to 34 years | 0.101 | 0.0500 | 0.0603 | 0.0669 | 0.0953 | 0.135 | 0.137 |
|  | (0.130) | (0.127) | (0.127) | (0.128) | (0.128) | (0.132) | (0.128) |
| 35 to 44 years | -0.164 | -0.258 | -0.140 | -0.137 | -0.122 | -0.0556 | -0.138 |
|  | (0.222) | (0.225) | (0.213) | (0.216) | (0.221) | (0.232) | (0.218) |
| 45 years or older | -0.0311 | -0.212 | 0.0623 | 0.0313 | -0.0141 | -0.0203 | 0.0375 |
|  | (0.253) | (0.243) | (0.249) | (0.250) | (0.252) | (0.255) | (0.251) |
| **Urban vs. rural (ref. = urban)** |  |  |  |  |  |  |  |
| Village | 0.0944 | 0.138 | 0.0570 | 0.0442 | 0.0804 | 0.119 | 0.0813 |
|  | (0.167) | (0.162) | (0.163) | (0.160) | (0.164) | (0.172) | (0.165) |
| **Gender (ref. = Male)** |  |  |  |  |  |  |  |
| Female | 0.417** | 0.339* | 0.403** | 0.407** | 0.447** | 0.450** | 0.429** |
|  | (0.153) | (0.151) | (0.150) | (0.151) | (0.152) | (0.159) | (0.151) |
| **Region (ref. = Central)** |  |  |  |  |  |  |  |
| Eastern | 0.187 | 0.199 | 0.112 | 0.231 | 0.195 | 0.135 | 0.211 |
|  | (0.168) | (0.161) | (0.163) | (0.167) | (0.168) | (0.173) | (0.167) |
| Northern | 0.736** | 0.822*** | 0.566* | 0.793*** | 0.751** | 0.774** | 0.737** |
|  | (0.232) | (0.233) | (0.223) | (0.222) | (0.228) | (0.239) | (0.228) |
| Western | 0.325 | 0.342 | 0.320 | 0.364 | 0.389 | 0.362 | 0.401 |
|  | (0.211) | (0.206) | (0.208) | (0.211) | (0.210) | (0.219) | (0.209) |
| **Marital status (ref. = never married)** |  |  |  |  |  |  |  |
| Divorced/separated/widowed | 0.872* | 1.007* | 1.029** | 0.934* | 0.957* | 0.986* | 0.998* |
|  | (0.413) | (0.428) | (0.394) | (0.394) | (0.398) | (0.423) | (0.402) |
| Married or living with partner | 0.116 | 0.152 | 0.132 | 0.150 | 0.121 | 0.0952 | 0.156 |
|  | (0.144) | (0.140) | (0.142) | (0.139) | (0.142) | (0.148) | (0.140) |
| **Highest education completed (ref. = completed primary)** |  |  |  |  |  |  |  |
| No formal schooling | 0.800 | 0.802 | 1.019** | 1.021* | 0.933* | 0.665 | 0.888* |
|  | (0.426) | (0.423) | (0.381) | (0.440) | (0.429) | (0.472) | (0.425) |
| Less than primary | -0.225 | -0.172 | -0.0993 | -0.153 | -0.189 | -0.211 | -0.212 |
|  | (0.232) | (0.243) | (0.226) | (0.224) | (0.227) | (0.240) | (0.230) |
| Completed secondary | -0.141 | -0.205 | -0.111 | -0.0826 | -0.131 | -0.127 | -0.106 |
|  | (0.179) | (0.170) | (0.174) | (0.177) | (0.177) | (0.184) | (0.176) |
| Completed tertiary | -1.067*** | -0.824*** | -0.934*** | -0.969*** | -0.994*** | -1.054*** | -0.994*** |
|  | (0.226) | (0.226) | (0.225) | (0.232) | (0.231) | (0.240) | (0.231) |
| **Main household income source before March 2020 (ref. = business income)** |  |  |  |  |  |  |  |
| Farming, livestock, or fishing | -0.273 | -0.285 | -0.146 | -0.165 | -0.206 | -0.224 | -0.273 |
|  | (0.171) | (0.175) | (0.165) | (0.168) | (0.168) | (0.176) | (0.169) |
| Remittances | -0.0915 | -0.139 | -0.151 | -0.130 | -0.0563 | -0.173 | -0.0466 |
|  | (0.367) | (0.338) | (0.361) | (0.374) | (0.360) | (0.397) | (0.364) |
| Wage-employment | -0.0259 | -0.206 | 0.0367 | -0.0374 | -0.00761 | -0.0343 | -0.0103 |
|  | (0.266) | (0.230) | (0.262) | (0.263) | (0.270) | (0.275) | (0.264) |
| Other sources | 0.720 | 0.693 | 0.669 | 0.738* | 0.631 | 0.692 | 0.677 |
|  | (0.369) | (0.376) | (0.375) | (0.369) | (0.365) | (0.360) | (0.363) |
| **Number of children < 18 in household (ref. = no children)** |  |  |  |  |  |  |  |
| 1 or 2 children | 0.324 | 0.294 | 0.262 | 0.317 | 0.349* | 0.296 | 0.305 |
|  | (0.171) | (0.163) | (0.166) | (0.166) | (0.168) | (0.173) | (0.166) |
| 3 or more children | 0.791*** | 0.894*** | 0.703*** | 0.688*** | 0.788*** | 0.770*** | 0.770*** |
|  | (0.177) | (0.176) | (0.173) | (0.179) | (0.178) | (0.181) | (0.175) |
| Constant | 3.722*** | 3.091*** | 3.053*** | 3.187*** | 4.105*** | 3.746*** | 3.714*** |
|  | (0.347) | (0.350) | (0.362) | (0.362) | (0.504) | (0.367) | (0.349) |
|  |  |  |  |  |  |  |  |
| **Observations** | 3722 | 3594 | 3760 | 3766 | 3801 | 3546 | 3808 |
| **Adjusted R-squared** | 0.056 | 0.101 | 0.089 | 0.067 | 0.053 | 0.053 | 0.051 |

Notes: Table shows output from Ordinary Least Squares (OLS) regressions of the Patient Health Questionnaire (PHQ)-4 on covariates including demographic characteristics and COVID-related variables. Each column represents a different regression, using a different COVID-related variable. Regressions used sampling weights. Robust standard errors are shown in parentheses.

**Table S7: Regression sensitivity analysis**

|  | (1) | (2) | (3) | (4) | (5) |
| --- | --- | --- | --- | --- | --- |
| Variables | OLS weighted | OLS unweighted | OLS weighted – without | NB RR weighted | Logit OR weighted PHQ4>6 |
|  |  |  |  |  |  |
| **Survey round (ref. = round 1)** |  |  |  |  |  |
| round = 2 | 0.35 | 0.20 |  | 1.07 | 1.09 |
|  | (0.27) | (0.17) |  | (0.06) | (0.20) |
| round = 3 | -0.03 | -0.06 | -0.00 | 0.99 | 0.93 |
|  | (0.25) | (0.16) | (0.25) | (0.05) | (0.16) |
| round = 4 | -0.05 | 0.00 | -0.06 | 0.99 | 0.98 |
|  | (0.25) | (0.16) | (0.24) | (0.05) | (0.17) |
| round =5 | 0.13 | 0.04 | 0.13 | 1.02 | 0.99 |
|  | (0.25) | (0.16) | (0.25) | (0.05) | (0.17) |
| **Gender (ref. = male)** |  |  |  |  |  |
| Female | 0.44*** | 0.20** | 0.46*** | 1.09*** | 1.40*** |
|  | (0.15) | (0.09) | (0.17) | (0.03) | (0.15) |
| **Urban vs. rural (ref. = urban)** |  |  |  |  |  |
| Village | 0.08 | 0.02 | -0.00 | 1.02 | 1.05 |
|  | (0.16) | (0.10) | (0.18) | (0.03) | (0.12) |
| **Long-term health problem (ref. = Do not have)** |  |  |  |  |  |
| You have | 0.33** | 0.32*** | 0.31* | 1.07** | 1.11 |
|  | (0.15) | (0.09) | (0.16) | (0.03) | (0.12) |
| **Age group (ref. = 18-24 years)** |  |  |  |  |  |
| 25 to 34 years | 0.12 | 0.14 | 0.10 | 1.02 | 1.17 |
|  | (0.13) | (0.11) | (0.14) | (0.03) | (0.11) |
| 35 to 44 years | -0.12 | -0.13 | -0.41* | 0.97 | 0.78 |
|  | (0.22) | (0.20) | (0.25) | (0.04) | (0.12) |
| 45 years or older | 0.02 | -0.00 | 0.06 | 1.00 | 0.99 |
|  | (0.25) | (0.24) | (0.28) | (0.05) | (0.18) |
| **Region (ref. = Central)** |  |  |  |  |  |
| Eastern | 0.20 | 0.04 | 0.24 | 1.04 | 1.19 |
|  | (0.17) | (0.11) | (0.19) | (0.04) | (0.14) |
| Northern | 0.74*** | 0.47*** | 0.86*** | 1.15*** | 1.71*** |
|  | (0.23) | (0.16) | (0.25) | (0.05) | (0.27) |
| Western | 0.40* | 0.27* | 0.39 | 1.08* | 1.29* |
|  | (0.21) | (0.15) | (0.24) | (0.04) | (0.19) |
| **Marital status (ref. = never married)** |  |  |  |  |  |
| Divorced/separated/widowed | 0.97** | 0.54** | 0.96** | 1.20** | 1.71** |
|  | (0.40) | (0.23) | (0.44) | (0.08) | (0.40) |
| Married or living with partner | 0.11 | 0.16 | 0.04 | 1.02 | 1.14 |
|  | (0.14) | (0.10) | (0.15) | (0.03) | (0.13) |
| **Highest education completed (ref. = completed primary)** |  |  |  |  |  |
| No formal schooling | 0.88** | 0.94*** | 1.19** | 1.17** | 2.26*** |
|  | (0.43) | (0.29) | (0.47) | (0.08) | (0.70) |
| Less than primary | -0.20 | -0.16 | 0.03 | 0.96 | 0.83 |
|  | (0.23) | (0.14) | (0.25) | (0.04) | (0.13) |
| Completed secondary | -0.12 | -0.17 | -0.12 | 0.98 | 0.87 |
|  | (0.18) | (0.11) | (0.19) | (0.03) | (0.11) |
| Completed tertiary | -1.01*** | -0.69*** | -0.79*** | 0.81*** | 0.53*** |
|  | (0.23) | (0.16) | (0.26) | (0.04) | (0.09) |
| **Main household income source before March 2020 (ref. = business income)** |  |  |  |  |  |
| Farming, livestock, fishing | -0.23 | -0.26** | -0.26 | 0.96 | 0.93 |
|  | (0.17) | (0.10) | (0.19) | (0.03) | (0.11) |
| Remittances | -0.05 | 0.15 | -0.06 | 0.99 | 0.93 |
|  | (0.36) | (0.24) | (0.39) | (0.07) | (0.23) |
| Wage-employment | -0.01 | -0.16 | 0.13 | 1.00 | 1.12 |
|  | (0.26) | (0.16) | (0.27) | (0.05) | (0.20) |
| Other sources | 0.67* | 0.50** | 0.68* | 1.14** | 1.35 |
|  | (0.36) | (0.25) | (0.38) | (0.07) | (0.37) |
| **Number of children < 18 in household (ref. = no children)** |  |  |  |  |  |
| 1 or 2 children | 0.32* | 0.20* | 0.46** | 1.07* | 1.19 |
|  | (0.17) | (0.12) | (0.19) | (0.04) | (0.15) |
| 3 or more children | 0.78*** | 0.67*** | 0.87*** | 1.17*** | 1.73*** |
|  | (0.17) | (0.13) | (0.20) | (0.04) | (0.24) |
|  |  |  |  |  |  |
| Constant | 3.84*** | 4.27*** | 3.81*** | 3.94*** | 0.33*** |
|  | (0.34) | (0.22) | (0.34) | (0.27) | (0.08) |
|  |  |  |  |  |  |
| Observations | 3,825 | 3,825 | 3,041 | 3,825 | 3,825 |
| R-squared | 0.06 | 0.03 | 0.06 |  |  |

Notes: Table shows output from Ordinary Least Squares (OLS) regressions of the Patient Health Questionnaire (PHQ)-4 on covariates including demographic characteristics and COVID-related variables. Each column represents a different regression, using a different COVID-related variable. Regressions used sampling weights. Robust standard errors are shown in parentheses.
